# Phase II study on the efficacy and safety of Lapatinib administered beyond disease progression and combined with vinorelbine in HER-2/*neu*– positive advanced breast cancer: results of the CECOG LaVie trial

**DOI:** 10.1186/s12885-016-2171-y

**Published:** 2016-02-18

**Authors:** Christiane Thallinger, Istvan Lang, Cvetka Grasic Kuhar, Rupert Bartsch, Christian F. Singer, Lubos Petruzelka, Bohuslav Melichar, Regina Knittelfelder, Thomas Brodowicz, Christoph Zielinski

**Affiliations:** Department of Medicine I and Comprehensive Cancer Center, Clinical Division of Oncology, Medical University Vienna – General Hospital, Währinger Gürtel 18-20, 1090 Vienna, Austria; Central European Cooperative Oncology Group (CECOG), Schlagergasse 6/6, 1090 Vienna, Austria; National Institute of Oncology, Ráth György u. 7-9, H-1122 Budapest, Hungary; Medical Oncology Department, Institute of Oncology Ljubljana, Zaloska 2, 1000 Ljubljana, Slovenia; Division of Gynecological Oncology, Währinger Gürtel 18-20, 1090 Vienna, Austria; Department of Oncology, Charles University Prague, U nemocnice 2, 12808 Prague, Czech Republic; Department of Oncology, Palacky University Medical School and Teaching Hospital, I.P. Pavlova 6, 77520 Olomouc, Czech Republic

**Keywords:** Metastatic Breast Cancer, HER-2/*neu*, Lapatinib, Vinorelbine, Chemotherapy

## Abstract

**Background:**

Vinorelbine constitutes effective chemotherapy for metastatic breast cancer (MBC) and acts synergistically with trastuzumab in HER-2/*neu* positive disease. The present study was set out to evaluate the efficacy and safety of vinorelbine when combined with lapatinib, an anti-HER2 tyrosine-kinase inhibitor, as late-line regimen administered beyond previous disease progression on prior lapatinib in patients with HER-2/*neu*- positive MBC.

**Methods:**

The CECOG LaVie study was designed as open-labeled, single-arm, multicenter phase II trial. Patients had to be pretreated with lapatinib plus chemotherapy, and received lapatinib at a daily dose of 1250 mg in combination with vinorelbine 20 mg/m^2^ i.v. on days 1 and 8 of a three-week cycle until disease progression, intolerable toxicity or withdrawal of consent. Progression-free survival (PFS) was defined as primary study endpoint; secondary endpoints included overall survival (OS), response rate according to RECIST 1.1, and safety. The study was terminated early due to poor accrual.

**Results:**

A total number of nine patients were included; lapatinib administered beyond disease progression combined with vinorelbine resulted in a median PFS of 7.7 months (95 % CI 0.56-14.91) and a median OS of 23.4 months (95 % CI 16.61–30.13), respectively. Partial remission was seen in one of nine patients, three patients had stable disease of > six months, whereas the remaining five patients had primary disease progression. In two patients, modification of vinorelbine dose due to toxicity became necessary; no dose modification was needed for lapatinib. The majority of reported adverse events (AE) were grade 1 and 2 in severity with diarrhea being the most commonly observed AE

**Conclusion:**

In this heavily pretreated patient population, combination of vinorelbine plus lapatinib showed encouraging activity and was characterized by an acceptable safety profile. Despite the low patient number, lapatinib plus vinorelbine may constitute a potential treatment option in heavily pretreated patients with HER-2/*neu-*positive MBC previously exposed to lapatinib.

**Trial registration:**

EudraCT number 2009-016826-15, (15. 10.2009)

## Background

Breast cancer is a heterogeneous disease encompassing distinct biological subtypes with distinct natural histories. The human epidermal growth factor receptor-2 (HER-2/*neu*) positive breast cancer subtype accounts for 10–15 % of all breast cancer cases. In the pre-trastuzumab-era, early-stage HER-2/*neu* positive disease had the second poorest prognosis after triple-negative breast cancer [1] thus leading to an accumulation of HER-2/*neu* positive breast cancers in the advanced disease setting [2, 3]. When highly expressed at membrane level, HER-2/*neu* undergoes hyperdimerization with itself or with other receptors of the erbB family thus activating various mitogenic pathways, specifically phosphatidylinositide 3-kinase (PI3K/AKT) and mitogen-activated kinase (MAPK) [4]. These pathways in terms induce continuous cell proliferation and new vasculature formation thus resulting in an aggressive phenotype with poor prognosis.

Trastuzumab, a monoclonal antibody targeting the HER-2/*neu* oncoprotein yields - in combination with various chemotherapies – significant and clinically relevant prolongation of progression-free survival (PFS) and overall survival (OS) as compared to chemotherapy alone. The varying interaction of trastuzumab with different chemotherapeutic drugs has been subject of various reports, which have indicated that such combination treatment might result in additive or even synergistic effects [5]. Of note, synergy was suggested for the combination of trastuzumab and vinorelbine, [6] which is popular due to its efficacy and ease of administration in conjunction with limited toxicity.

Despite high activity, resistance to trastuzumab will eventually occur during the course of treatment in metastatic HER-2/*neu*-positive disease, which has led to the development of various alternative anti-HER2 compounds including tyrosine-kinase inhibitors (TKIs) such as lapatinib and second-generation antibodies such as pertuzumab or T-DM1.

As member of the 4-anilinoquinazoline classes of kinase inhibitors, lapatinib reacts with the ATP binding site of EGFR, and HER2/*neu* thus resulting in inhibition of autophosphorylation and subsequent proliferative signaling [7]. Lapatinib is currently approved for the treatment of patients with HER-2/*neu* positive MBC who progressed on prior trastuzumab-based therapy or as first-line treatment in combination with letrozole in luminal B/HER-2/*neu* positive disease [8].

Treatment of MBC relies upon cascade-like sequential administration of cytotoxic compounds, thereby offering a chance for prolonged disease control [9–11]. In HER-2/*neu* positive breast cancer, a prolongation of PFS by the continuation of trastuzumab beyond progression under the proviso of a change of the hitherto administered cytotoxic drug was demonstrated (treatment in multiple-lines; TML) [12].

Vinorelbine constitutes effective chemotherapy for metastatic breast cancer (MBC) and acts synergistically with trastuzumab in HER-2/*neu* positive disease.

Based upon these considerations, it seemed reasonable to hypothesize that the combination of lapatinib plus vinorelbine could also result in significant anti-tumor activity in the challenging setting of late-line treatment in patients with HER-2/*neu* positive MBC pretreated with trastuzumab and lapatinib. Indeed, encouraging activity of lapatinib plus vinorelbine combination therapy has been already demonstrated in two phase II trials [13, 14] while the concept of lapatinib beyond disease progression was not investigated henceforth.

Thus, the objective of this phase II trial was to assess activity and safety of lapatinib plus vinorelbine in HER-2/*neu* positive patients with MBC who had progressed on previous lapatinib-based treatment.

## Methods

### Study design

This multicenter, open-labeled, single arm phase II trial included female HER-2/*neu* positive patients with MBC. Patients were enrolled between October 2010 and August 2012 from 7 sites in 4 countries.

All eligible patients with MBC were pretreated with lapatinib in combination with various cytotoxic drugs excluding vinorelbine. Lapatinib was administered beyond disease progression and prescribed at a dose of 1250 mg p.o. once daily on a continuous basis. Vinorelbine was administered at a dose of 20 mg/m^2^ by intravenous infusion on days one and eight of a three-week cycle until disease progression or the necessity of discontinuation of study treatment due to unacceptable toxicity, withdrawal of consent, loss to follow up, or death.

The primary aim was to evaluate PFS in heavily pretreated MBC patients receiving the combination of lapatinib and vinorelbine with a descriptive intent only.

### Patient population

Eligible patients were women ≥18 years of age with histologically or cytologically confirmed HER2/*neu* positive (HER2/*neu* 3+ as defined by immunohistochemistry and/or *HER-2/neu* gene amplification as defined by fluorescence in situ hybridization) MBC with at least one measureable lesion according to Response Evaluation Criteria in Solid Tumors version 1.1 (RECIST v.1.1). Prior lapatinib-based treatment in first- or second-line therapy for metastatic disease was mandatory. Patients were required to have adequate organ and bone marrow function, Eastern Cooperative Oncology Group performance status of 0–1, life expectancy of more than 12 weeks and an adequate left ventricular ejection function of at least 50 % at baseline, as measured by either echocardiography or MUGA scan.

Exclusion criteria included concomitant endocrine therapy, previous radiotherapy for metastatic disease in order to allow for appropriate bone marrow reserve within frame of the current trial, active cardiac, hepatic or biliary disease and diseases or surgeries affecting gastrointestinal function. Patients undergoing concurrent treatment with anticancer or investigational agents, females pregnant or lactating, and those with a peripheral neuropathy of grade 2 or greater were also excluded. Any medication that was considered necessary for the patient’s welfare and was not expected to interfere with the evaluation of study treatment could be given at the discretion of the investigator. Other antitumor therapies were not permitted.

The study was approved by independent ethics committees and was conducted in accordance with the principles of the Declaration of Helsinki and the Note of Guidance on Good Clinical Practice. All patients provided written informed consent prior to study entry.

The study was approved by the following ethics committees: Ethikkommission des KH der Elisabethinen, Ethikkommission des Landes Salzburg, Ethikkommission der Medizinsiche Universität Wien, Etická komise FN Olomouc, Etická komise Všeobecné fakultní nemocnice, Medical Research Council Ethics Committee for Clinical Pharmacology, National Medical Ethics Committee of the Republic of Slovenia.

### Study endpoints

Baseline tumor assessment (CT or MRI) of the chest, abdomen and brain were performed within 28 days before first study drug application and once every 6 weeks during treatment phase thereafter. Tumor response data were assessed by the investigator according to RECIST criteria v.1.1. Additionally, a bone scan was mandatory at baseline.

The primary endpoint was progression free survival (PFS), defined as the time from study entry until the first observation of disease progression according to the above schedule or death due to any cause. Secondary endpoints included overall survival (OS), defined as the time from study entry until death, objective response rate (ORR), defined as the percentage of patients experiencing confirmed complete response (CR) and partial response (PR) assessed by RECIST criteria v.1.1, and safety.

### Safety and Tolerability

Safety parameters included adverse events (AEs) and serious AEs (SAEs), hematology and clinical chemistry, physical examination, periodic measurements of vital signs and electrocardiograms (ECGs) and evaluation of changes of left ventricular ejection fraction (LVEF). AEs were coded using the Medical Dictionary for Regulatory Activities (MedDRA) and assessed according to National Cancer Institute Common Terminology Criteria for Adverse Events (NCI-CTCAE version 4.0).

### Statistical analysis

For this pilot study, sample size was not based on statistical considerations and therefore no formal sample-size calculation was conducted. A sample size of thirty patients was planned initially were considered to be appropriate for a phase II study in order to gain information on efficacy and safety of the study treatment in this setting. No interim analysis was planned. Study endpoints are provided above.

The study was closed due to slow accrual in April 2014 and data of the nine patients who were included into the study were analyzed. Descriptive statistical methods were used to summarize the study results.

## Results

### Patient characteristics

In total, nine female patients with locally recurrent, inoperable, or metastatic MBC were enrolled. All nine patients - representing the intention-to-treat (ITT) population – received at least one dose of study medication.

Median patient age was 51,66 years (range 37–75 years).

All patients were tested HER-2/*neu* positive by IHC or FISH analyses, and six patients had endocrine dependent disease (luminal B / Her2/*neu*; four patients had estrogen/progesterone receptor positive cancer, one patient estrogen receptor positive/progesterone receptor negative and one patient estrogen receptor negative/progesterone receptor positive).

### Prior systemic treatment

All patients were heavily pretreated.

Six patients had received anthracycline-based regimens in the neoadjuvant or adjuvant setting, whereas all patients had received prior capecitabine and seven taxanes (six patients docetaxel, one patient paclitaxel) for MBC.

One patient received prior lapatinib as third-line treatment for metastatic disease, which was not according to protocol.

Individual patients and treatment characteristics are given in Table 1.

**Table 1:** Individual patients and treatment characteristics

Patient number	1	2	3	4	5	6	7	8	9
Menstrual status	Premenopausal	Postmenopausal	Premenopausal	Premenopausal	Postmenopausal	Premenopausal	surgically sterilized	Postmenopausal	Premenopausal
ECOG performance status	1	0	1	0	0	0	0	0	0
Hormone receptor status (PR/ER)	PR pos/ER pos	PR pos/ER pos	negative	PR pos/ER pos	PR pos/ER pos	negative	negative	PR pos/ER neg	PR neg/ER pos
Metastatic at initial diagnosis	No	No	Yes	No	No	No	No	No	Yes
Number of metastatic sites									
Target lesions	4	5	1	2	1	3	1	2	1
Non-target lesions	1	3	1	2	2	2	2	2	1
Site of metastatic disease	Liver	Liver	Lung	Lung	Lung	Lymphnodes	Lung	Thoracic wall	Liver
	Bone	Lung	Liver	Lymphnodes		Bone		Skin	Bone
Prior neo-adjuvant treatment	Yes	No	No	No	No	Yes	Yes	Yes	No
Prior adjuvant treatment	No	Yes	No	Yes	Yes	Yes	No	No	No
Prior metastatic treatment									
Endocrine therapy	first line	No	No	No	No	No	No	No	first line
Chemotherapy	second line	first/second/third line	first/second/third line	first/second line	first/second line	first/second line	first/second line	first line	first/second/third line
Trastuzumab	first line	first line	first line	first line	first line	first line	No	first line	first line
Lapatinib	second line	second line	second line	second line	second line	second line	first line	second line	third line

### Treatment exposure

In total, 74 cycles were delivered to 9 patients (range 2–19 cycles). Three patients showed remarkable long treatment duration and received 14, 15 and 19 treatment cycles, respectively. Lapatinib dose of 1250 mg/day was maintained in all patients without any dose reduction needed. Vinorelbine dose was reduced from 20 mg/m^2^ to 12 mg/m^2^ in two patients due to toxicity.

### Efficacy

Over the study period, six out of nine patients had died due to disease progression or disease-related complications. Median PFS was 7.7 months (95 % CI 0.56–14.91), and median OS 23.4 months (95 % CI 16.61–30.13).

One patient experienced partial remission, three patients had stable disease over > six months, whereas the remaining five patients had disease progression at first restaging.

The three patients with longest treatment showed the longest PFS of 10.0, 12.8 and 14.6 months, respectively: Two of them had stable disease and one of them was the only partial responder on this study. No particular characteristics discerning these three patients from the others were identified.

### Safety

In total, 117 adverse events have been reported in this study. The relationship to study drug was balanced between lapatinib and vinorelbine (18.8 % related to lapatinib, 19.7 % related to vinorelbine and 15.4 % related to both). The majority of the reported adverse events (*n* = 105) were grade 1 and 2 in severity.

One patient experienced two grade 4 adverse events (hyperbilirubinemia and elevated level of GGT). For this patient, elevated liver enzymes have been described in the medical history at study entry and both grade 4 events considered to be unrelated to study treatment by the investigator.

One patient died due to grade 5 pulmonary embolisms, which was rated by investigator to be unrelated to study treatment.

Nine grade 3 adverse events were reported in 5 patients including neutropenia, diarrhea, hepatotoxicity, elevated levels of liver enzymes, hyponatremia, hypertension and humerus fracture.

Adverse events in this study were consistent with the known lapatinib and vinorelbine safety profiles.

Three serious adverse events have been reported (humerus fracture, pulmonary embolism and hypertension), all of them were considered unrelated to study treatment.

Most common adverse events are summarized in Table 2.

**Table 2:** Most common adverse events

	Grade 1/2 n (%)	Grade 3 n (%)	Grade 4 n (%)	Total n (%)
Diarrhea	20 (17.1)	1 (0.9)	0 (0)	21 (17.9)
Neutropenia	9 (7.7)	1 (0.9)	0 (0)	10 (8.5)
Leucopenia	8 (6.8)	0 (0)	0 (0)	8 (6.8)
Increased alkaline phosphatase	3 (2.6)	1 (0.9)	0 (0)	4 (3.4)
Nausea	4 (3.4)	0 (0)	0 (0)	4 (3.4)
Fatigue	4 (3.4)	0 (0)	0 (0)	4 (3.4)

## Discussion

Treatment of metastatic cancer is characterized by the onset of treatment resistance, which leads to tumor progression and ultimately to the patients’ death. To a degree, this course can be extended by the cascade-like use of different primarily efficacious, yet ultimately ineffective drugs. The spectrum of such drugs differs according to the molecular and biologic pattern of MBC. In patients with HER-2/*neu* positive MBC who are in the focus of the present report, available drugs include trastuzumab and lapatinib and, recently added, pertuzumab [15] and T-DM1 [16]. Of note, resistance to trastuzumab-based treatment can be partially overcome by maintaining trastuzumab beyond disease progression [12] while switching chemotherapy, which is known to interact with trastuzumab in a divergent manner according to the used substance [5]. Ultimately, however, activity of trastuzumab is lost requiring alternative treatment approaches. It was this widely therapy-resistant patient population with HER-2/*neu* positive tumors we had in mind when the present protocol for the LaVie study was designed. For this pilot study, sample size was not based on statistical considerations and therefore no formal sample-size calculation was conducted. In this study design trastuzumab- and lapatinib-pretreated patients with HER-2/*neu* positive MBC were planned to be treated with lapatinib beyond progression with the addition of vinorelbine. The latter compound was chosen as chemotherapy backbone due to first, its previously described synergistic activity with trastuzumab [6] and second, the fact that many patients with HER-2/*neu* positive disease would be ultimately treated with lapatinib and capecitabine [17] without any evidence-based further treatment option.

With encouraging results from phase I trials testing for the combination of lapatinib and vinorelbine available [18, 19], the present data in a heavily pretreated patient population show that lapatinib plus vinorelbine constitutes another safe and moderately efficacious treatment option for HER-2/*neu* positive MBC. The trial was terminated due to poor recruitment, which can be explained by its design, which foresaw the inclusion of patients with very late line of treatment. The problem is reflected well by the fact that six out of nine patients died during the study period due to progression of their respective disease or to disease-related complications. Thus, the data obtained in this small population of patients have to be interpreted with caution. Nevertheless, even in this heavily pretreated population at late disease stage, the combination of lapatinib and vinorelbine resulted in a median PFS of 7.7 months (95 % CI 0.56–14.91) and a clinical benefit in four out of nine patients with HER-2/*neu* MBC who had either partial remission (one patient) or stable disease of > six months duration (three patients). Of note, results with regards to PFS are similar to PFS data from the lapatinib registration trial; in this prospective randomized phase III trial, the combination of lapatinib plus capecitabine yielded 8.4 months median PFS [17]. Thus, our results indicate that lapatinib-based therapy beyond disease progression may be feasible in a population of women with MBC who had received both, lapatinib and capecitabine previously.

Although naturally limited by the small number of patients accrued, data from the present study indicate that similar to trastuzumab, administration of lapatinib in multiple-lines may constitute a meaningful treatment option in selected patients and vinorelbine is valuable cytotoxic combination partner for lapatinib. Since the initiation of the LaVie trial, several novel anti-HER2 agents became available. It is therefore unlikely that the concept of lapatinib-based treatment in multiple lines will be investigated in larger studies. Therefore, and with all caution due to the mentioned limitations, we believe that results of LaVie add knowledge to the field or therapy of HER2/*neu* metastatic breast cancer.

## Conclusion

The present study was set out to evaluate the efficacy and safety of vinorelbine when combined with lapatinib, an anti-HER2 tyrosine-kinase inhibitor, as late-line regimen administered beyond previous disease progression on prior lapatinib in patients with HER-2/*neu*- positive MBC. In this heavily pretreated patient breast cancer population, combination of vinorelbine plus lapatinib showed encouraging activity and was characterized by an acceptable safety profile. Despite the low patient number, lapatinib plus vinorelbine may constitute a potential treatment option in heavily pretreated patients with HER-2/*neu-*positive MBC previously exposed to lapatinib.
